# Migration of guiding catheter during placement of hemodialysis femoral catheter: case report

**DOI:** 10.11604/pamj.2023.44.27.35662

**Published:** 2023-01-12

**Authors:** Marouane Jabrane, Abdelkarim Kharroubi, Chaima Cherrad, Badr Elkassimi, Mohamed Arrayhani

**Affiliations:** 1 Department of Nephrology and Kidney Transplantation, Hassan II Hospital, Faculty of Medicine and Pharmacy, Ibn Zohr University, Agadir, Morocco,; 2 Department of Vascular and Endovascular Surgery, Hassan II Hospital, Faculty of Medicine and Pharmacy, Ibn Zohr University, Agadir, Morocco

**Keywords:** Catheter, migration, hemodialysis, case report

## Abstract

Migration of guiding catheter during placement of hemodialysis femoral catheter is an unusual, early and rare mechanical complication. We report here the case of a 70-year-old man, admitted for severe renal failure, uremic syndrome and hyperkalemia, requiring an extra renal purification session which was complicated by a blockage of the femoral venous catheter guide during its removal. Such a complication highlights the importance of good anatomical knowledge, good monitoring by an experienced person during central venous catheterization, and the interest in using ultrasound guidance before and after catheter placement.

## Introduction

Central venous catheterization is an essential tool in nephrology; it constitutes the vascular access that can be used for urgent indications of hemodialysis or following the temporary or definitive loss of the functionality of an arteriovenous fistula. However, the use of the catheter remains burdened with potentially severe mechanical, infectious, thrombotic or hemorrhagic complications. The femoral approach is more frequently complicated by infection and thrombosis than by mechanical complications [1]. We report here the case of a migration of femoral catheter guide during its removal to start hemodialysis session.

## Patient and observation

**Patient information**: a 70-year-old man was referred to the emergency department for management of severe renal failure with 2400 umol/L of creatinine and dialysis was not successful on right femoral catheter. He had a medical history combining hypertension under treatment, and a correct vaccination status against COVID-19.

**Clinical findings**: the patient was admitted to a COVID-19 unit due to the appearance of symptoms and a positive polymerase chain reaction (PCR) test. The patient was then referred to the emergency room, since he has hyperkalemia at 7.8meq/L, with a uremic syndrome. The indication for hemodialysis with placement of a central venous catheter was retained. The right femoral approach was chosen. A 20 cm arrow 12G double lumen catheter was inserted, after two attempts, without ultrasound guidance, there was no venous return and a blockage when removing the guide was noted. Following this, the patient was referred to our hospital for further management. The examination on admission revealed a conscious man with a Glasgow coma score of 15/15^th^, hypertensive to 150/77 mmHg, slightly discolored conjunctiva, good pulsed oxygen saturation at 97% under 10 liters of oxygen in a high concentration mask, polypneic to 32 cycles/min. The biological assessment showed severe renal failure (urea= 3.21 g/L, creatinemia= 272 mg/L with creatinine clearance at 6 ml/min). On physical examination, the patient had no hematoma on the inner side of the right thigh and no abnormal bleeding. Hemodialysis was therefore indicated and a catheter was inserted in the left femoral vein without complications. At D1 of hospitalization in COVID-19 unit, the patient presented a pelvic pain with swelling of the right inner thigh.

**Figure 1:**
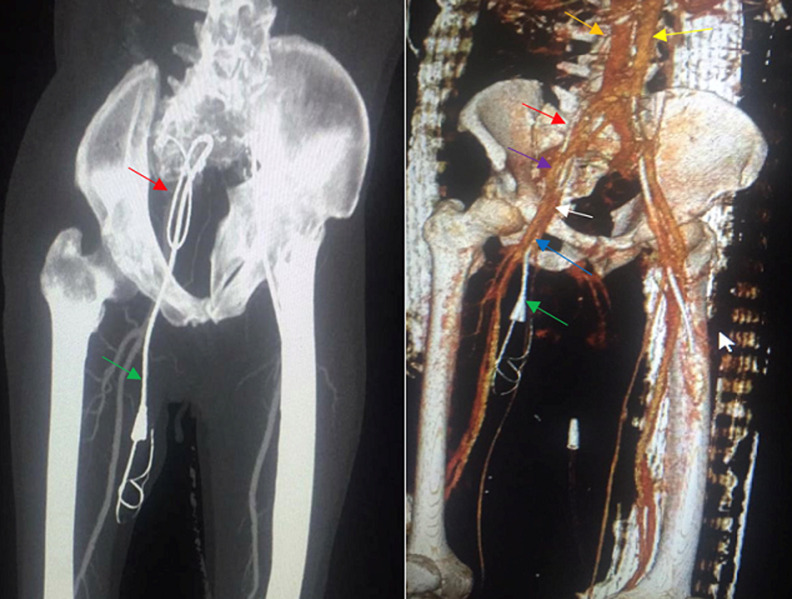
scanning image with 3D reconstruction: right femoral catheter crossing the vascular wall of the right femoral vein and looped in the psoas muscle with migration of the guide out of the vein path (red arrow)

**Diagnostic assessment**: on the 2^nd^ day of its hospitalization, an emergency angioscan of the lower limbs was performed, which revealed a right femoral catheter crossing the vascular wall and guide migration in the right femoral vein and looping the right psoas muscle (Figure 1).

**Therapeutic intervention**: the patient has benefited from removal of the right femoral catheter by a vascular surgeon without hemorrhagic complications. The evolution was clinically favorable in a few days.

**Informed consent**: written informed consent was obtained from the patient for publication of this case report and accompanying images.

**Patient perspective**: “I was surprised by this complication which remains very rare according to the explanations of the doctor, and I hope that the sharing of my case helps to improve the management of similar patients”.

## Discussion

In nephrological emergencies, when hemodialysis is necessary, the placement of a central venous catheter, in particular a femoral venous catheter, is recommended because it´s easy to access and allows a good perfusion flow, but this procedure presents a high risk of infectious and thrombotic complications [2]. Knowledge of infectious, thrombotic, hemorrhagic and mechanical complications is of great importance [3]. At the femoral site, mechanical complications are rare essentially dominated by hematoma and arterial puncture, some authors have reported exceptional complications of central venous catheterization, iatrogenic ileal perforation reported by El Bouazzaoui *et al*. [4], late vascular perforation in the inferior vena cava territory reported by Gil *et al*. [5], and finally, the case of migration of the metal stent in the lumen of the femoral vein reported by Berrada *et al*. [6]. In our case, blockage of the femoral catheter guide and its migration to the psoas muscle is an early and rare mechanical complication, never described in the literature, to our knowledge. This complication could have been avoided by a good knowledge of the anatomical landmarks, good monitoring by an experienced person, and by the use of ultrasound guidance during placement of the femoral vein catheter.

The femoral vein is usually punctured at Scarpa's triangle, below the crural arch stretched between the anterior superior iliac spine and the pubic spine. Placement of the femoral venous line is performed by percutaneous puncture, in a blind fashion, aided by knowledge of the usual anatomy of the vessels, skin and bone landmarks, and palpation of the nearby femoral artery [2]. The experience of the surgeon plays a major role in the occurrence of mechanical complications. Sznajder *et al*. demonstrates in a study that the overall failure rate of central venous catheter placement was 10.1% for experienced physicians and 19.4% for inexperienced physicians. Proportionally, the complication rate was 5.4%/11% [7]. Also Eisen *et al*., demonstrate that the rate of mechanical complication increased with the number of percutaneous punctures, with a rate of 54% when more than 2 punctures were necessary [8].

The use of ultrasound guidance has been promoted as a method to reduce the risk of complications and failure during central venous catheterization, to make the procedure safer and to improve patient comfort [9]. In this technique, an ultrasound probe is used to locate the vein and measure its depth under the skin. Under ultrasound visualization, the introduction needle is then guided through the skin and into the vessel. Like all imaging techniques, ultrasound guidance requires training. In hospitals where ultrasound equipment is available and physicians have adequate training, the use of ultrasound guidance should be systematically considered for cases where femoral and internal jugular vein catheterization will be attempted [10]. According to a meta-analysis by the National Institute for Health and Care Excellence (NICE), recommendations have been issued, highlighting up the necessity of using ultrasound for any catheter placement [11]. In our case, we believe that the absence of ultrasound and the insertion of the catheter with several attempt, contributed to increasing the risk of mechanical complications in particular migration and blockage of the femoral venous catheter guide.

**Ethical statement**: the research was conducted in accordance with the principles embodied in the [2] and in accordance with local statutory requirements. An informed consent was obtained from the patient.

## Conclusion

The placement of a central venous catheter, particularly the femoral venous catheter, should be reserved for emergencies and situations where other routes are not possible. Through this observation, we can say that this procedure can be complicated if it is not done in the recommended standards. The prevention of these complications requires the respect of anatomical landmarks, the use of ultrasound guidance during the placement of the central venous catheter, as well as a perfect mastery of the technique by the actor.
